# A Bacterial Myeloperoxidase with Antimicrobial Properties

**DOI:** 10.3390/biotech12020033

**Published:** 2023-05-05

**Authors:** Claire Céré, Brigitte Delord, Parfait Kenfack Ymbe, Léa Vimbert, Jean-Paul Chapel, Claire Stines-Chaumeil

**Affiliations:** CNRS, University of Bordeaux, CRPP, UMR5031, 115 Avenue Schweitzer, F-33600 Pessac, France

**Keywords:** myeloperoxidase, heme, hypohalide and pseudo-hypohalide products, microbicidy

## Abstract

The four mammalian peroxidases (myeloperoxidase, eosinophilperoxidase, lactoperoxidase, and thyroid peroxidase) are widely studied in the literature. They catalyze the formation of antimicrobial compounds and participate in innate immunity. Owing to their properties, they are used in many biomedical, biotechnological, and agro-food applications. We decided to look for an enzyme that is easiest to produce and much more stable at 37 °C than mammalian peroxidases. To address this question, a peroxidase from *Rhodopirellula baltica*, identified by bioinformatics tools, was fully characterized in this study. In particular, a production and purification protocol including the study of heme reconstitution was developed. Several activity tests were also performed to validate the hypothesis that this peroxidase is a new homolog of mammalian myeloperoxidase. It has the same substrate specificities as the human one and accepts I^−^, SCN^−^, Br^−^, and Cl^−^ as (pseudo-) halides. It also exhibits other auxiliary activities such as catalase and classical peroxidase activities, and it is very stable at 37 °C. Finally, this bacterial myeloperoxidase can kill the *Escherichia coli* strain ATCC25922, which is usually used to perform antibiograms.

## 1. Introduction

**Figure 1:**
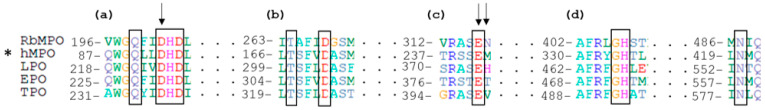
Sequence alignment of four mammalian peroxidases with *Rhodopirellula baltica* peroxidase. (**a**,**c**) Essential amino acids of the distal heme cavity site. (**b**) Essential amino acids of the Ca^2+^ binding site. (**d**) Essential amino acids of the proximal heme cavity site. The amino acid numbering of * hMPO corresponds to the numbering in the PDB 1CXP. The three arrows correspond to the residues involved in the covalent bond with heme. The dashed arrow corresponds to the sulfonium bond only present in hMPO.

Heme peroxidases are ubiquitous in all organisms, such as plants, mammals, yeast, and bacteria. The different families are described on the RedOxiBase website (https://peroxibase.toulouse.inra.fr/, access on 1 January 2020) [1]. Mammalian peroxidases (MMPs) belong to the cyclooxygenase peroxidase superfamily. The four MMPs—human myeloperoxidase (hMPO), eosinophilperoxidase (EPO), lactoperoxidase (LPO), and thyroid peroxidase (TPO)—are characterized by the presence of a prosthetic heme group covalently bound to the active site. HMPO possesses two ester bonds and one sulfonium link with heme through D94, E242, and M243, respectively. In contrast, EPO, LPO, and TPO contain only two ester bonds between conserved aspartate and glutamate in their sequence alignment (Figure 1) [2,3,4]. HMPO is found in neutrophils and plays a role in innate immunity [5,6]. It is involved in the host defense mechanism to fight microbicidal attacks [7,8]. For example, hMPO is bound to the neutrophil extracellular trap in the macrophage suicide mechanism [9]. EPO is also involved in innate immunity and is localized in eosinophil granulocytes [10]. LPO is found in mucous membranes, saliva, tears, and milk. It plays a role in disinfection and protection against microorganisms [10,11]. TPO is a membrane-bound enzyme, found in the thyroid gland. It is involved in the synthesis of the thyroid hormone [12]. The maturated forms of these enzymes present several post-translational modifications. For example, hMPO possesses light and heavy chains, intra- and inter-chain disulfide bridges, and glycosylations [13,14,15]. The number and type of covalent bonds with heme influence substrate specificity [16]. The bound sulfonium present in hMPO is responsible for chlorination activity compared to the low activity in EPO and the lack of activity with this halide in LPO. Depending on the MMPs, the substrate specificity is as follows: (i) hMPO and EPO: SCN^−^ > I^−^ > Br^−^ >> Cl^−^; (ii) LPO: SCN^−^ > I^−^ >> Br^−^ [17,18]. These peroxidases react with H_2_O_2_ to form compound I (Fe^IV^ = O+porphyrin**^·^**). As mentioned above, they have several (pseudo-) halides as substrates to catalyze the reduction of compound I into the native form (Fe^III+^ porphyrin) [19,20]. The hMPO also has classical peroxidase activity as well as superoxidase and catalase activity [13,21].

Among the heme peroxidases of bacterial origin, LsPPOx, a peroxidase from *Lyngbya* sp. has been extensively studied [2,22] and shows strong similarities with LPO. It exhibits substrate specificity I^−^ > SCN^−^ > Br^−^ and no activity with Cl^−^. On the other hand, nothing is known about bacterial heme peroxidase that has similarities to hMPO and EPO, including chlorination activity.

These enzymes have antimicrobial and virucidal activities [8,23,24,25,26,27,28]. They are of interest and are used in various biomedical or biotechnological fields. MPO porcine is used in E-101 solutions to disinfect human or veterinary injuries [29]. LPO is used in lactoperoxidase system for milk conservation and in food processing [30]. To operate these systems, glucose oxidase is currently used to provide H_2_O_2_ continuously in the presence of its two substrates, glucose, and O_2_ [31].

From the sequence database and primary sequence analysis (Figure 1), a peroxidase from *Rhodopirellula baltica* (RbMPO) was identified as a putative bacterial homolog of mammalian peroxidase. The essential amino acids corresponding to the distal and proximal heme cavity or binding site for Ca^2+^ are conserved. The objective of this study was to prove experimentally that RbMPO is a bacterial counterpart of mammalian peroxidases, easy to produce, stable, and with microbicidal properties for use in future biomedical applications. In this work, we cloned, produced in *Escherichia coli*, purified from inclusion bodies, reconstituted with its prosthetic heme group, and biochemically, enzymatically, and functionally characterized RbMPO. The major results are the broad and extensive activities covered by this enzyme. It combines classical peroxidase activity toward 2,2’-azino-bis(3-ethylbenzothiazoline-6-sulfonic acid (ABTS), and also produces hypohalides and pseudohypohalides from NaCl, NaI, NaBr, or NaSCN and exhibits catalase activity at pH 7.5. In addition, we also presented the pre-steady-state analysis of the formation of compound I. We highlighted its potential application to control microbes and described the experimental setting using a protocol derived from the inhibitor at a minimal concentration on the typical ATCC 25922 strain used for antibiotic experiments.

## 2. Materials and Methods

### 2.1. Materials

Oligonucleotides were synthesized by Sigma. Plasmid sequencing was performed by Genewiz (Leipzig, Germany). Substrates used for RbMPO activity were: NaSCN from Sigma > 99.99%, NaBr from Sigma > 99.99%, and NaCl from Sigma BioXtra > 99.5%. Lysogenic Broth (LB) was from MP Biomedicals™ and Tryptic Soy Broth (TSB) was from Becton Dickinson. Ninety-six-well plates were from Greiner bio-one. Triton X-100, 4-aminobenzoic acid hydrazide (ABAH), Aminophenylfluorescein (APF), and absolute ethanol were both from Sigma. HAZ-TABS 4.5 g of chlorine (Guest Medical) was used diluted in water as a disinfectant. Catalase was from bovine liver (Sigma) and *E. coli* strain 25922 was from ATCC. Isopropyl b-D-1-thiogalactopyranosil (IPTG) was from Euromedex. ABTS was from Roche.

### 2.2. Experimental Procedures

#### 2.2.1. Genetic Constructions

The open reading frame (ORF) RbWH47_01796 was amplified by polymerase chain reaction (PCR) using the genomic deoxyribonucleic acid (DNA) from *R. baltica* WH47. Genomic DNA was purified with DNeasy^®^ Blood and Tissue from biomass kindly provided by Dr Jens Harder. Genomic DNA was used as a template and the forward and reverse primers were 5′GCCAACGTTGTTGCACATCATATGTTGTTCTGGTCG 3′ and 5′GCTTCTACGGTGCCGAACTCGAGAGCGAGGACG 3′, respectively. The forward primer contains a *Nde*I restriction site and the reverse primer contains an *Xho*I restriction site. These restriction sites were used to clone RbWH47_01796 into a plasmid derived from pET21a, yielding pET21aRbMPO-6His (tag in C-term) for overexpression under the control T7 promotor. The cloned RbWH47_01796 ORF was sequenced to confirm that no mutations were introduced in the amplification reaction. The primers 5′TTTATCGTCCTCGCTTGAGAGCACCACCACCAC 3′.

5′GTGGTGGTGGTGCTCTCAAGCGAGGACGATAAA 3′ were used to insert a stop codon before the tag in the sequence. The Quick-Change mutagenesis kit (Stratagene) was used.

#### 2.2.2. Production and Purification of RbMPO

The recombinant plasmid for the expression of the tagless RbMPO was transformed into *E. coli* BL21Star (DE3). An overnight culture was used to inoculate 1L of LB supplemented with 100 µg/L ampicillin and 35 µg/L chloramphenicol. The culture was incubated at 37 °C until it reached the exponential growth phase (0.6–0.8 A_600nm_), then protein expression was induced with 500 µM IPTG followed by 24 h incubation at 22 °C. Cells were harvested by centrifugation at 6000× *g* for 20 min at 4 °C, then the pellet was washed with 50 mM 2-Amino-2-hydroxymethyl-propane-1,3-diol (TRIS) pH 7.5 buffer, supplemented with antiprotease tablet (Roche), and crushed twice (TS2 0.75kW Serie Bench Top, Constant systems Ltd., Daventry, United-Kingdom) at 2200 bar/4 °C. Pellets were sonicated for 10 min and incubated in a urea solution (8 M urea, 20 mM TRIS pH 7.5, 100 mM NaCl) for 4 h under stirring at 4 °C. Another centrifugation step at 21,000× *g*/4 °C/45 min was required, and the supernatant underwent successive dialysis steps (6 M > 4 M > 2 M > 0 M of urea) every 12 h. After centrifugation at 21,000× *g*/4 °C/45 min and filtration at 0.45 µm, the protein solution was injected onto a Hiload^®^ 16/60 Superdex^®^ 75pg (Cytiva) previously equilibrated with 50 mM TRIS pH 7.5 and 5.5 mM CaCl_2_ (buffer A). Isocratic elution with the same buffer as equilibration allowed us to collect RbMPO in the dead column volume (size of RbMPO: 78,272 Da and column splitting domain: 3000–70,000 Da). The enzymatic fraction was diluted in buffer A and injected in a Resource™ Q 6 mL (Cytiva) pre-equilibrated with 50 mM TRIS pH 7.5, 5.5 mM CaCl_2_, and 1 M NaCl (buffer B). The apo RbMPO was eluted with an increasing gradient of buffer B (0–1 M NaCl). To obtain the holo-RbMPO, an additional step of reconstitution overnight was performed. Apo RbMPO was incubated with hemin 50 µM, CaCl_2_ 5.5 mM, TRIS 0.1 M, oxidized glutathion 0.75 mM, and then incubated for 24 h on a wheel at 4 °C. Purified enzymes were freeze-dried and stored at 4 °C after ultrafiltration and diafiltration against a 50 mM pH 7.5 TRIS buffer without any trace of chloride. All purification steps were taken using an Äkta system (GE Healthcare). The enzyme concentration was determined spectrophotometrically, using the theoretical epsilon of RbMPO: ε = 49,850 M^−1^·cm^−1^ and absorbance at 280 nm.

#### 2.2.3. Site Titration of RbMPO by Heme

The Trp fluorescence quenching experiment was performed in a spectrofluorometric FP8300 with a temperature-controlled stirred cell (25 °C). Increasing concentrations of heme were used (0–60 µM) and the fluorescence intensity of apo RbMPO was measured at 450–300 nm by exciting the sample first at 297 nm and then at 334 nm as a function of time. Sample dilutions were performed in a 50 mM pH 7.5 sodium phosphate buffer.

#### 2.2.4. Inhibition of the Enzyme by ABAH

The following range of ABAH was used: 0, 0.5, 0.1, 0.25, 0.5, 0.6, 0.7, 0.8, 0.9, 1, 5, 50, 100, 500, and 1000 µM. Fixed concentrations of NaCl, H_2_O_2_, enzyme, and APF were used: 500 mM, 500 µM, 1 µM, and 10 µM, respectively. Fluorescence emission was measured at 525 nm (λex = 485 nm). Measurements were performed in Corning 384-well clear-bottom (flat-bottom) plates with a reaction volume of 20 µL. Each measurement was performed in triplicate at 37 °C and in 50 mM pH 7.5 sodium phosphate buffer. A duration of 10 min kinetic was performed for each value of the ABAH range. The measured fluorescence was proportional to the quantity of fluorescein, which in turn is proportional to the quantity of Cl^−^ produced by RbMPO (the fluorescence intensity is compared to the standard fluorescein range).

#### 2.2.5. Regeneration of RbMPO under Native Form by Various Salts

20 µM of H_2_O_2_ and salts (100 µM NaI, 1 mM NaBr, 500 µM NaCl or 100 µM NaSCN) were rapidly mixed with 1.79 µM of RbMPO with a Biologic SFM400 stopped flow at 412 nm, 37 °C and with 50 mM pH 7.5 sodium phosphate buffer under single turnover conditions. The recovery of the absorbance at 412 nm after an initial decay step due to the formation of compound I was monitored until the enzyme returned to the absorbance at 412 nm corresponding to the native state.

#### 2.2.6. Steady-State Kinetic Parameters of RbMPO

Initial rate measurements under steady-state conditions were performed with a BioLogic SFM400 stopped-flow using a single-mixing mode and a Xe/Hg lamp and a TC100/10 cell. Table 1 summarizes the different conditions of assays performed on RbMPO. One syringe was filled with RbMPO and the other one with or without taurine, H_2_O_2_, and NaBr or NaSCN, or NaCl. The appearance of taurine bromamine (Tau-NHBr), taurine chloramine (Tau-NHCl), and hypothiocyanate (^−^OSCN) was measured at 289, 253, and 240 nm, respectively at 37 °C in the presence of a 50 mM pH 7.5 sodium phosphate buffer. At least five 120 s shots were monitored for each value of the substrate range with the following sampling rates: 20 µs, 20 ms, and 20 ms with 2000 points per period. Slopes were calculated from the average shots for each concentration and *k*_ss_ (s^−1^) were determined from the slopes using the following formula:

$$k_{ss}=\frac{slope\;\left(Abs.s^{-1}\right)}{\varepsilon_{product}\;\left(M^{-1}.cm^{-1}\right)}\frac{1}{\left[RbMPO\right]\left(M\right)}\frac{1}{l\;\left(1cm\right)}$$

RbMPO concentrations in the assay were 300 nM for NaBr and NaSCN and 2 µM NaCl, respectively, and molar extinction coefficients for each product were ε_Tau-NHBr_ = 415 M^−1^·cm^−1^, ε_Tau-NHCl_ = 429 M^−1^·cm^−1^ and ε_-OSCN_ = 951 M^−1^·cm^−1^ [22,32]. The collected data were analyzed with Origin software, then saturation curves for each substrate were fitted either by non-linear regression with or without excess substrate inhibition (Equations (1) and (2), with [S] the substrate of interest, or by linear regression (Equation (3)) with *a* the slope and *b* the y-intercept.

(1) $$k_{ss}=\frac{k_{cat}*\left[S\right]}{K_{M}+\left[S\right]}$$

(2) $$k_{ss}=\frac{k_{cat}*\left[S\right]}{K_{M}+\left[S\right]*\left(1+\frac{\left[S\right]}{K_{i}}\right)}\;$$

(3) $$k_{ss}=k_{2}*\left[S\right]+b$$

The effect of temperature on the activity of RbMPO for each of its (pseudo-)halogenated substrates was studied using a range from 10 to 60 °C at 267 nM RbMPO with 5 mM SCN^−^ or 500 mM Br^−^ and at 1.78 µM RbMPO with 500 mM Cl^−^. The H_2_O_2_ concentration was 79 µM.

The effect of pH was tested at 267 nM RbMPO with SCN^−^ or Br^−^ or 1.78 µM RbMPO with 100 mM Cl^−^ in various buffers at the same ionic strength as described by Su et al. [33].

#### 2.2.7. Stability of RbMPO at 4 and 37 °C

The stability of RbMPO was measured for up to 30 days. RbMPO was stored in a 50 mM TRIS buffer pH 7.5 at two temperatures, namely 4 °C and 37 °C. The enzyme was concentrated at 123.4 μM for the 4 °C storage condition and at 132.2 μM for the 37 °C storage condition. The MPO activity of the enzyme was assessed at different time points by a standard activity test. To do so, the enzyme was diluted at 1 µM and contacted with H_2_O_2_ (79 µM), NaCl (500 mM), and APF (10 µM). The measured fluorescence was proportional to the quantity of fluorescein, which in turn is proportional to the quantity of Cl^−^ produced by RbMPO (the fluorescence intensity is compared to the standard fluorescein range).

#### 2.2.8. Catalase Activity

Catalase activity was measured using a stopped-flow setup. The instrument was configured to measure absorbance decay at 240 nm (see Table 1) (ε_H2O2_ = 43.6 M^−1^·cm^−1^) and 37 °C. A range of H_2_O_2_ was performed with 2 µM of RbMPO: 0, 0.02, 0.05, 0.1, 0.25, 0.5, 0.75, 1, 2, 5, 10 mM in a 50 mM pH 7.5 sodium phosphate buffer.

#### 2.2.9. Peroxidase Activity

ABTS was used as a substrate for RbMPO to measure peroxidase activity. Measurements were performed in a 384-well plate (Corning^®^ 384 well microplate) at 37 °C and each condition was performed in triplicate. Fixed concentrations of RbMPO and H_2_O_2_ were used (0.5 nM and 79 µM, respectively) in a final volume of 20 µL in 50 mM sodium phosphate buffer pH 5.5. The plate reader was configured to run a 10 min kinetics at 420 nm (ε_ABTS_._+_ = 36 mM^−1^·cm^−1^, see Table 1) with the following ABTS range: 0, 0.1, 0.2, 0.5, 1, 2, 5, 10, 15, 20, 30 and 40 mM.

#### 2.2.10. Microbicidal Effect on *E. coli* ATCC 25922

*E. coli* 25922 was grown on TSB agar for 16 h at 37 °C. One colony was grown in 10 mL of TSB medium under stirring at 190 rpm at 37 °C. The optical density (OD) of the bacterial preculture was measured at 620 nm in a spectrophotometer (Ultrospec 10, Biochrom). A total of 25 mL of TSB medium were inoculated to obtain an OD of approximately 0.09. When the OD was between 1 and 2, a dilution in 10 mL of TSB medium was performed to obtain 2 × 10^6^ colony forming unit (CFU) per mL. Two tests were performed, one by following growth kinetics in a microplate and another by counting colonies on a Petri dish. For the first test, bactericidal tests were performed in 96-well microplates under a final volume of 100 µL, with 10^6^ CFU/mL. The plate cover was treated with 0.05% TritonX-100 and 20% ethanol to prevent condensation and, therefore, errors in reading the wells by the instrument. The solution was allowed to act on the lid for 10 min, then removed and dried under a class II biological safety cabinet. Each tested condition was performed at least in triplicate and each experiment was repeated at least three times. The kinetics was set to 16 h with a measurement at 620 nm every 15 min in a microplate reader (Wallac Victor 21420 Multilabel counter) thermostated at 37 °C [shaking duration: 10.0 s; shaking speed: fast; shaking diameter: 0.5 mm; shaking type: orbital; measurement time: 0.2 s; numbers of replicates: 60; time between replicates: 900 s; label: P620]. Enzymes essays were performed: first with glucose oxidase from *Aspergillus niger* (GOx) alone, then RbMPO alone, and finally with the coupled enzymatic system. For all these experiments, a substrate such as NaSCN and glucose (for GOx only or the coupled enzymatic system) were at fixed concentrations, respectively, at 25 mM and 15 mM (glucose present in the culture media). A total of 5 mM bleach was used as a positive control and bacteria alone was used as a negative control. For the test on the Petri dish, the bacterial culture at 1 × 10^6^ CFU/mL was then contacted with the enzyme solutions for 1 h (in a final volume of 1 mL). Further dilutions were then made: 10^−3^, 10^−4^, 10^−5^, 10^−6^. A total of 100 µL of these solutions were spread on a Petri dish containing TSB agar medium and placed at 37 °C overnight. The next day, the surviving colonies were counted, and the number of CFU/mL was calculated using the formula below:

$$\frac{CFU}{mL}=\frac{Nb_{colony}*10}{dilution\;}$$

The experimental conditions were 0 or 25 nM of GOx, 0 or 300 nM of RbMPO, and 25 mM of NaSCN.

## 3. Results

### 3.1. Production and Purification of RbMPO

**Figure 2:**
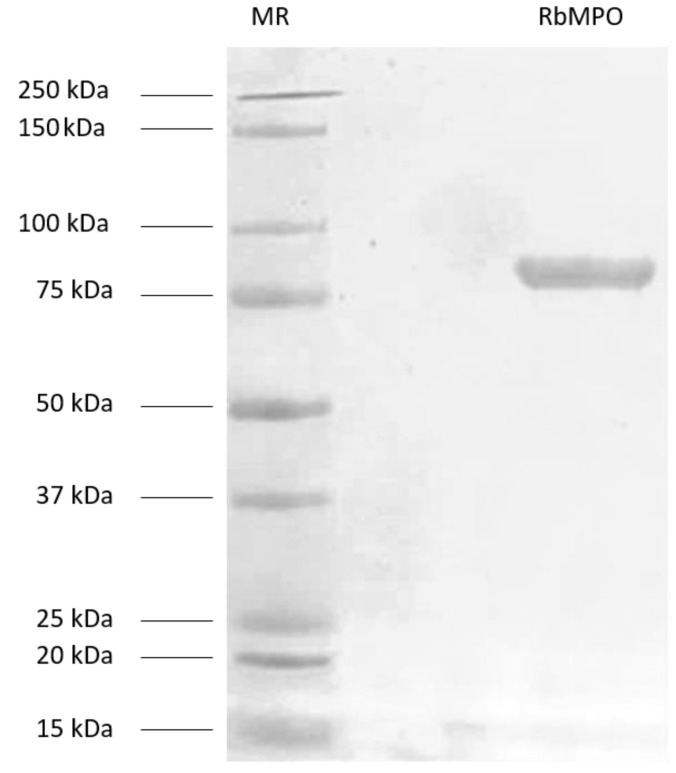
Gradient 4–15% SDS-PAGE. MR: Mass Ruler and RbMPO concentrate after purification.

**Figure 3:**
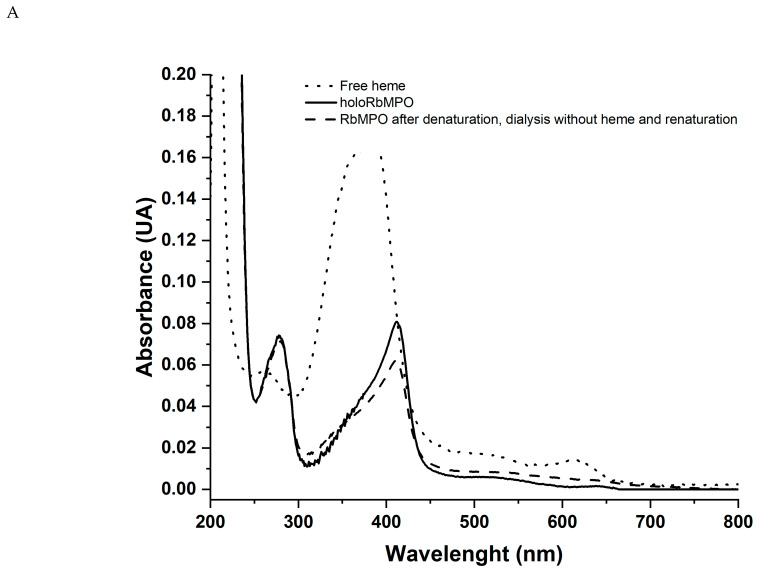

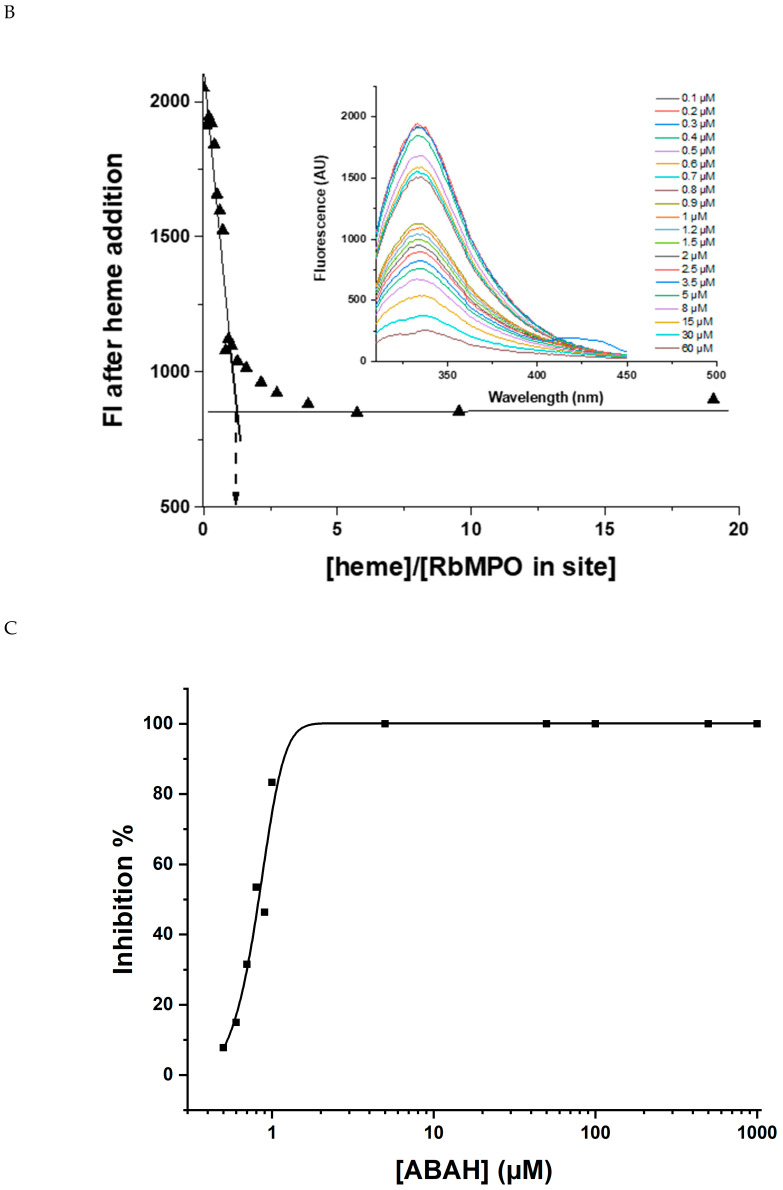
(**A**) UV-visible spectrum of RbMPO at 1.46 µN in site before and after denaturation/renaturation without the addition of heme in the dialysis bath and free heme; (**B**) Titration plot of apo RbMPO by heme from emission spectrum obtained from the plot in inset after W excitation at 297 nm; (**C**) Effects of ABAH on the chlorination activity of RbMPO. Fixed concentrations of enzyme and substrates were used: 1 µN in the site of RbMPO, 500 mM of NaCl, 500 µM of H_2_O_2,_ and 10 µM of APF. Each measurement was performed in triplicate at 37 °C and in 50 mM pH 7.5 sodium phosphate buffer. IC50 was of 0.80 ± 0.09 by applying the dose-response following equation: % of inhibition (i.e., activity without ABAH corresponds to 100% of the activity or 0% of inhibition and activities measured with various concentrations of ABAH corresponds to x% of the activity or (100 − x)% of inhibition of myeloperoxidase activity) = bottom + ((Top-Bottom)/(1 + (10^LogIC50^/10 [ABAH])^Hillslope^); with bottom = 0 ± 23; top = 100 ± 3 and Origin 2019: 9.6.0.172.

As shown in Figure 2, RbMPO is of sufficiently pure quality to perform enzymatic assays. This protocol allows us to obtain about 30 mg of purified enzyme from 2 L of bacterial culture. The enzyme was produced under apo form without heme. Consequently, it was not possible to follow the activity at each step of the purification process. The UV-visible spectrum shows two different peaks. The peak at 280 nm is characteristic of tyrosine and tryptophan amino acids, which are components of RbMPO. Heme reconstitution of the enzyme was demonstrated by the presence of a Soret band at 412 nm as shown on the RbMPO spectrum (Figure 3A). The Reinheitszahl (R_z_) ratio (A_412nm_/A_280nm_) of RbMPO is 0.98 ± 0.11 depending on the batch. To test if the heme was linked or not by a covalent bond in the RbMPO, the peroxidase was denatured with urea 8 M then the same renaturation protocol as described for the folding of enzymes present in the inclusion bodies was used. UV-visible spectrum (Figure 3A) of holo-RbMPO before and after unfolding/folding showed that the heme was not lost during the denaturation step and dialysis and the Rz was 0.89 ± 0.02.

### 3.2. Reconstitution of the Active Site of RbMPO

**Figure 4:**
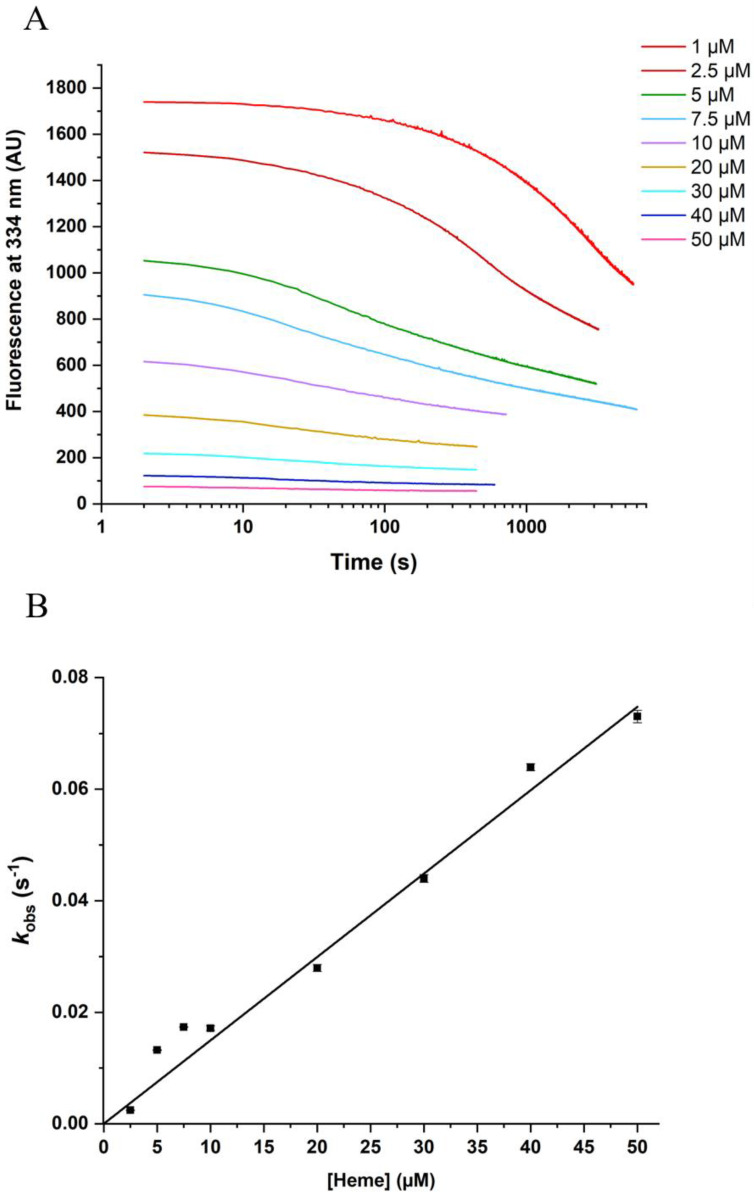
(**A**) Fluorescence kinetics at 334 nm after excitation at 297 nm observed after the addition of various concentrations of heme on RbMPO at 1 µN in site (**B**) Graphical representation of the reconstitution rate constants (*k*_obs_) from the fits of the mono-exponential function to the experimental data shown in A as a function of heme concentration. *k*_2_ corresponding to the slope is 1500 ± 241 M^−1^·s^−1^.

Scanning measurements (450–300 nm) after excitation at 297 nm showed a fluorescence peak at 330 nm. An increase in heme concentration resulted in a quenching of the fluorescence intensity at this peak (inset of Figure 3B). This means that there is a conformational rearrangement in the 3D structure, which is represented by a decrease in tryptophan availability followed by quenching of tryptophan fluorescence emission. These experiments allowed us to observe the transition of the enzyme from the apo to the holo form. Analysis of fluorescence intensity at 330 nm for each heme concentration provided a site titration with one molecule of heme bound per monomer of RbMPO (Figure 3B). Kinetic experiments at 330 nm after excitation at 297 nm permit the following of a slow conformational rearrangement during heme binding to RbMPO (Figure 4A). The mono-exponential analysis of each kinetic allowed us to determine *k*_obs_ values at each concentration of heme and represented a slow rearrangement following the heme entrance efficiency of RbMPO. RbMPO has an environment that favors efficient reconstitution with heme, the increase of *k*_obs_ was proportional to the concentration of heme and the *k*_2_ was 1500 ± 241 M^−1^·s^−1^ (Figure 4B).

### 3.3. Inhibition of the Enzyme by ABAH

ABAH is known to be an irreversible inhibitor of native hMPO [34]. Since we assumed that hMPO and RbMPO have a similar function, we expected ABAH to also act as an inhibitor of the bacterial enzyme. To determine whether ABAH influences RbMPO, measurement of the chlorination activity of the enzyme was performed in the presence of a range of ABAH. The appearance of fluorescein was measured at 525 nm, indicating the formation of the enzyme reaction product, HOCl, which had reacted with the APF probe. Figure 3C shows the inhibition percentage of chlorination activity of RbMPO. The activity of the enzyme is inhibited as the concentration of ABAH increases. IC50 was 0.80 ± 0.09 µM. At 5 µM ABAH, 100% of RbMPO activity is inhibited. This molecule can then be considered to be an inhibitor of the bacterial enzyme.

### 3.4. Pre-Steady-State Kinetic Parameters of RbMPO

**Figure 5:**
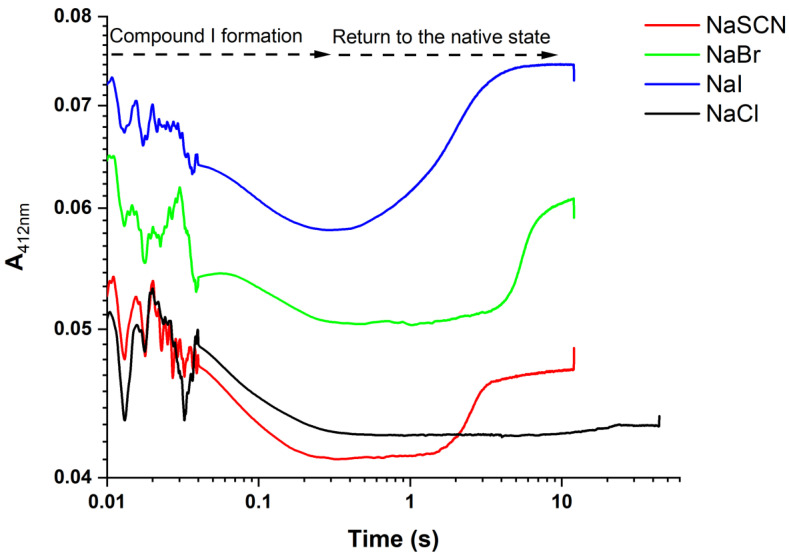
Kinetic traces obtained after rapid mixing of RbMPO 1.78 µN in site with H_2_O_2_ 20 µM in the presence of NaSCN or NaI at 100 µM, NaBr at 1 mM, or NaCl at 500 µM at 37 °C.

The formation of compound I of RbMPO was studied under single turnover conditions by rapid kinetics analysis in the presence of various salts (Figure 5). The transition of the enzyme from native to compound I form results from the oxidation of heme Fe^III^ to Fe^IV^. The heme Soret band is at 412 nm for the native Fe^III^ iron form, so we followed the decrease in absorbance at this particular wavelength as a function of time. The return of the enzyme to its native state was studied by the addition of the (pseudo)-halide substrates on compound I. Kinetics at 412 nm allowed us to highlight the formation of compound I with an exponential decrease in absorbance followed by a return to the initial A_412nm_ value when there is not enough H_2_O_2_ to maintain the enzyme in the form of compound I; typical curves obtained in the presence of NaSCN, NaI, NaCl or NaBr are shown in Figure 5. The time to return to the native state was, respectively, around 3, 4, 25, and 10 s at 100 µM NaSCN or NaI, 500 µM of NaCl or 1 mM of NaBr, and at 20 µM of H_2_O_2_ in measuring cell, respectively.

### 3.5. Steady-State Kinetic Parameters of RbMPO

#### 3.5.1. Halogenation Activity

The stopped flow was used to define the steady-state kinetic parameters of RbMPO. For each (pseudo)-halogenated substrate, a concentration range was performed at fixed concentrations of enzyme and H_2_O_2_. The appearance of the enzymatic product was measured at different wavelengths depending on the substrate: at 240 nm with direct measurement for ^−^OSCN formation, while at 253 nm and 289 nm, Tau-NHCl or Tau-NHBr were measured, respectively (see Table 1). From the averages of the curves obtained for each shot, the slopes in Abs·s^−1^ were calculated and then converted into *k*_ss_ by the following relation:

$$k_{\mathrm{ss}}=\frac{slope}{\varepsilon_{-OX}}\;\frac{1}{\left[RbMPO\right]}$$

Kinetic parameters were summarized in Table 2. Saturation was observed for NaSCN or NaBr allowing the determination of the Michaelis constant (*K*_M_), respectively, of 0.51 ± 0.08 mM and 133 ± 32 mM, and catalytic rate constant (*k*_cat_), respectively, of 56.9 ± 6.92 s^−1^ and 76.3 ± 5.12 s^−1^. No saturation was observed for NaCl and *k*_2_ was determined to be 5.46 ± 0.67 M^−1^·s^−1^. The substrate specificity for RbMPO was SCN^−^ > Br > Cl^−^.

#### 3.5.2. Catalase Activity

The measurement of the decrease in absorbance at 240 nm allowed us to characterize the catalase activity of RbMPO. At pH 7.5, RbMPO showed catalase activity with the steady-state rate constant (*k*_ss_) versus H_2_O_2_ concentration curve showing substrate inhibition. *K*_M_ and *k*_cat_ were 0.17 ± 0.11 mM and 56.6 ± 20.6 s^−1^, respectively. The *k*_cat_/*K*_M_ ratio was of 3.33 × 10^5^ ± 3.36 · 10^5^ M^−1^·s^−1^ (Table 2).

#### 3.5.3. Peroxidase Activity

*k*_ss_ values were found to increase linearly with ABTS concentration with a *k*_2_ value of 1.06 × 10^3^ ± 0.15 × 10^3^ M^−1^·s^−1^ (Table 2).

### 3.6. Microbicidal Activity

**Figure 6:**
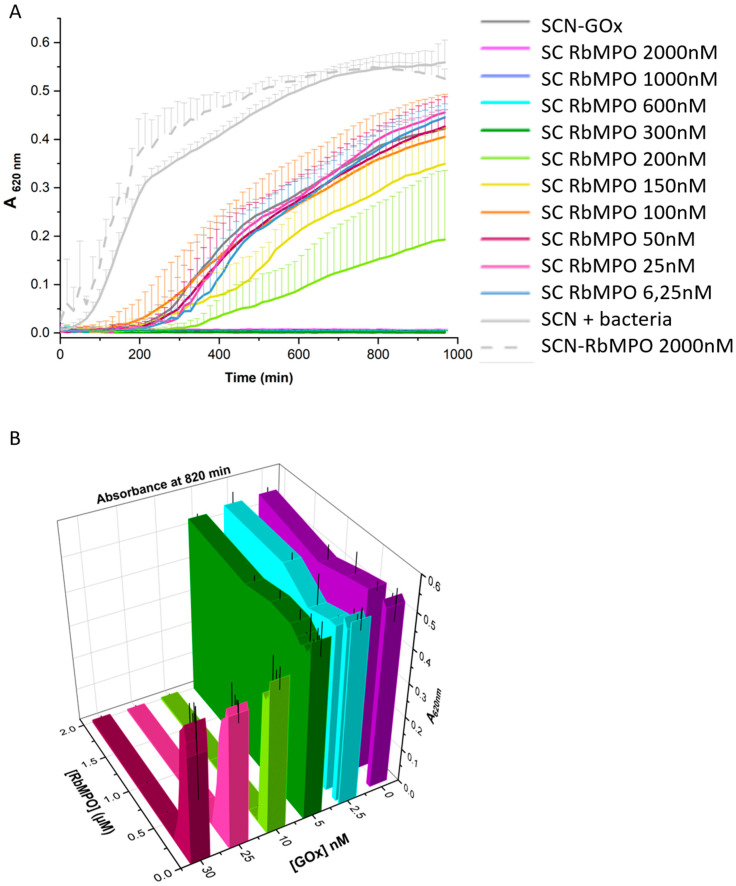
Bactericidal activity of RbMPO in the presence of 25 mM of SCN^−^ and glucose oxidase as H_2_O_2_ supplier. (**A**): Example of an A_620nm_ measurement in a 16 h kinetic with 25 nM of GOx and a range of RbMPO. CS: coupled system. (**B**): A_620nm_ at 820 min for each tested condition (range of GOx and RbMPO).

**Figure 7:**
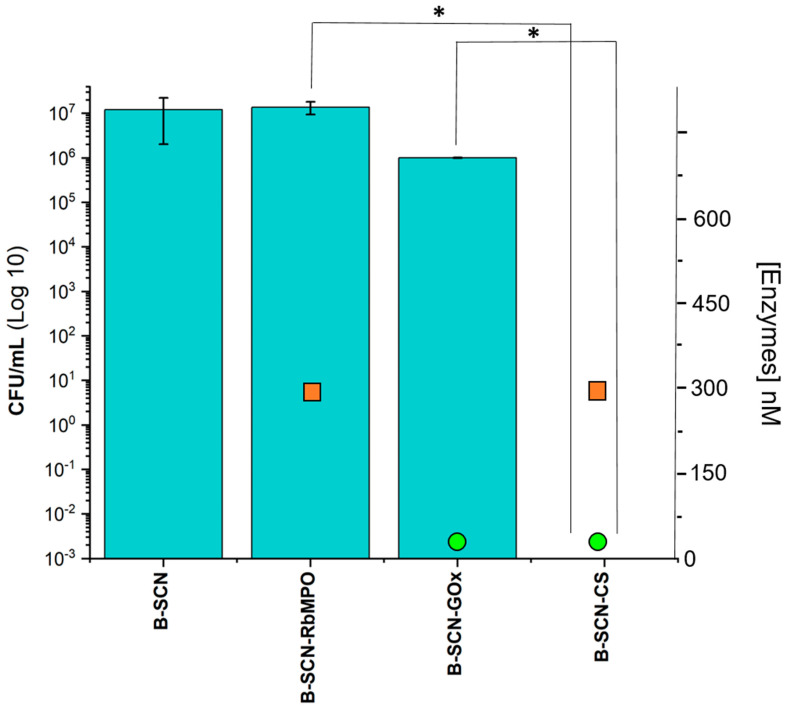
Histogram summarizing the different tested conditions in the presence of 1.10^6^ CFU/mL (noted B- on the *x*-axis) and in contact for 1 h with the enzymatic solution at pH 7.4. The CFU/mL defined on the ordinate axis (in log 10) corresponds to the number of surviving bacteria after one night at 37 °C in the incubator. The 2nd ordinate axis corresponds to the enzyme concentration (RbMPO alone, GOx alone, or coupled system) present in the incubation solution. * means that the difference is statistically significant with a *p*-value less than 0.05.

The first method to measure the activity of the RbMPO product was to monitor the growth of the ATCC25922 *E. coli* strain in the presence or absence of several concentrations of the two enzymes that compose the coupled system: the GOx and the RbMPO. GOx was used as a supplier of H_2_O_2_ and RbMPO as a catalyst to make the pseudo-hypohalogenous compound ^−^OSCN. The coupled enzymatic system works continuously thanks to the glucose present in the culture medium of the strain and the H_2_O_2_ continuously produced by the GOx. Figure 6A shows an example of kinetics obtained in the presence of 25 nM GOx, increasing concentrations of RbMPO, and 25 mM of NaSCN. The graph shows that the higher the concentration of RbMPO, the more bacterial growth is delayed until complete inhibition. Results obtained with various concentrations of GOx and various concentrations of RbMPO are presented in Figure 6B under 3D graphs. The results show that a minimum ratio of 10:1 between the concentration of RbMPO and GOx was required to completely inhibit growth and lead to a 100% bactericidal effect over 16 h of kinetics. The second method to demonstrate the bactericidal effect of RbMPO is the CFU counting technique that is commonly used in pharmacopeia to test the effect of antibiotics on microorganisms (Figure 7). *E. coli* ATCC 25922 bacteria were incubated for 1 h in solutions containing the enzymes and substrate required for ^–^OSCN production: 25 mM of NaSCN, 25 nM of GOx, and 300 nM of RbMPO at pH 7.4. There was no significant difference in colony number between bacteria with substrate ± RbMPO or between bacteria with substrate ± GOx with a p-value, respectively, of 0.85 or 0.26. For the coupled enzymatic system, complete inhibition of bacterial growth was observed. As in the 96-well plate experiment, 25 mM SCN^−^ in the presence of 25 nM GOx and 300 nM RbMPO is sufficient to kill 100% of the bacteria (*p*-values of 0.0002 with “GOx alone” and 0.048 “RbMPO alone”). Here, only a one-hour incubation between the 1.10^6^ CFU/mL bacterial dilution and the coupled system solution was sufficient to inhibit bacterial growth.

### 3.7. Activity in Function of Temperature, pH, and Storage Conditions

**Figure 8:**
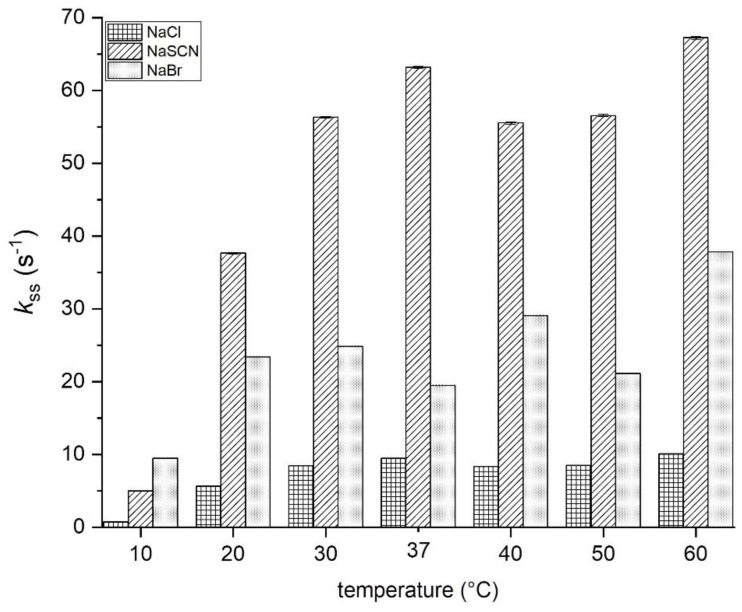
Effect of temperature on the formation of halogenate or pseudo-halogenate compounds by the RbMPO enzyme. Measurements were carried out at 240, 253, and 289 nm for ^−^OSCN, Tau-NHCl, and Tau-NHBr, respectively, at 37 °C in a 50 mM pH 7.5 sodium phosphate buffer in the presence of 267 nM RbMPO for assays with 5 mM SCN− or 500 mM Br^−^ and of 1.78 µM RbMPO for assays with 500 mM Cl^−^. The concentration of H_2_O_2_ was 79 µM in all assays. Each condition has a minimum average of 5 points. *Origin 2019: 9.6.0.172*.

**Figure 9:**
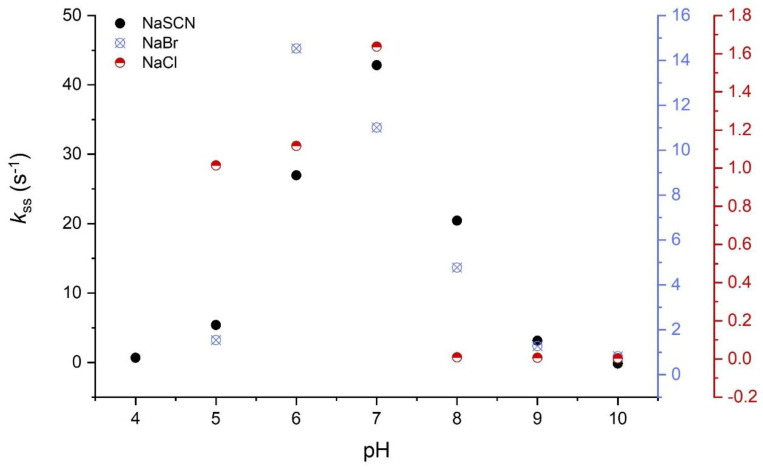
Effect of pH on the formation of halogenate or pseudo-halogenate compounds by the RbMPO enzyme. Measurements were performed at 240, 253, and 289 nm for ^−^OSCN, Tau-NHCl, and Tau-NHBr, respectively, at 37 °C in different buffers at fixed ionic strength as described by Su et al. [33] at 267 nM of RbMPO mixed with 5 mM SCN− or 100 mM Br^−^ or at 1.78 µM of RbMPO mixed with 100 mM Cl^−^. Each condition has a minimum average of 5 points. *Origin 2019: 9.6.0.172*.

**Figure 10:**
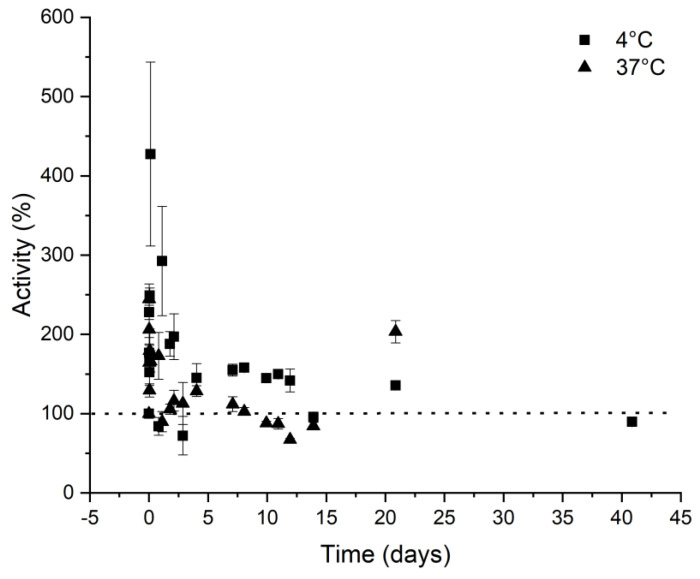
Activity of RbMPO (i.e., chlorination activity measured with APF fluorescent probe, which reacts with HOCl if the enzyme has produced it) stored in a TRIS 50 mM buffer pH 7.5 at 4 °C (^▄^) and 37 °C (▲), for up to 30 days. The myeloperoxidase activity was measured at 525 nm after excitation at 485 nm at 37 °C, using standard assay (1 µM RbMPO, 37 °C in 50 mM sodium phosphate at pH 7.5, 500 mM NaCl, 79 µM H_2_O_2_ and 10 µM APF).

RbMPO maintained its activity over a wide range of temperatures, i.e., from 20 to 60 °C during the activity test and with the three salts tested (NaCl, NaBr, and NaSCN) as shown in Figure 8. A decrease in activity was observed at 10 °C with the three salts. Figure 9 shows the activity of RbMPO as a function of pH and at fixed ionic strength. The optimum pH is 7 for NaCl and NaSCN as substrates and 6 for NaBr. Chlorination activity was observed between pH 5 and 7, and bromination activity and formation of hypothiocyanate between pH 5 and 9 was observed with residual activity with NaSCN at pH 4. RbMPO is very stable at 4 and 37 °C in 50 mM TRIS buffer at pH 7.5. At least 40 days for the RbMPO stored at 4 °C and at least 20 days for the RbMPO stored at 37 °C (Figure 10).

## 4. Discussion

A robust production protocol, two-step chromatographic purification, and reconstitution of RbMPO have been established. Approximately 30 mg of pure enzyme was obtained per 2 L of culture. The various enzyme reconstitution tests show that the optimum is at pH 7.5 in 50 mM TRIS for 24 h in the presence of a large excess of heme. A complementary study of the fluorescence quenching of RbMPO shows that the enzyme has one heme per monomer. A slow conformational rearrangement was also observed after the addition of heme to the enzyme. Monitoring this rearrangement in the presence of increasing heme concentrations indicates an absence of saturation over the concentration ranges tested. After reconstitution of a purified enzyme, the Rz was around 1. In our case, this ratio and the fluorescence quenching of tryptophan assays indicate that the heme was bound to the enzyme but do not indicate the purity index as might be the case for the native enzyme produced directly in the soluble fraction and already reconstituted with their prosthetic group before purification. In this study, the enzyme was first purified and then reconstituted with heme. An alternative would be to find strategies to increase the proportion of soluble RbMPO already reconstituted with heme before purification. However, it was interesting to have the protein in the apo form to study the reconstitution step. The first results here show that heme was not released after the denaturation of holo-RbMPO, which means that the heme was probably covalently bound to the active site. A further step will be to analyze by site-directed mutagenesis the role of key amino acids involved in the binding of heme in the active site. The amino acid E316 and D201 are conserved in sequence alignment comparing mammalian peroxidase and RbMPO and could be certainly important for the covalent binding of heme. Mutagenesis experiments will be further considered. The chlorination activity of RbMPO was studied in the presence of increasing concentrations of ABAH. The result shows that the enzyme is inhibited by this molecule which is a well-known inhibitor of hMPO as described by Kettle et al. [34] or Furtmüller et al. [35]. These results support our hypothesis that the active site of hMPO and RbMPO are very similar. In the hMPO model and in the presence of H_2_O_2_, hMPO changes from its native state to compound I. ABAH reacts with compound I to form ABAH· and compound II. This form of the FeIV enzyme reacts with another molecule of ABAH to return to the native state and produce a second ABAH·. Finally, the native enzyme reacts with ABAH· to regenerate ABAH and convert hMPO into inactive compound III form. The irreversible inhibition lies in the ability of H_2_O_2_ and ABAH to cause the release of heme from the active site of the enzyme by destroying the covalent bonds responsible for the maintenance of the prosthetic group in the active site of the enzyme [34,35]. Further studies will be performed on RbMPO to verify the model described for hMPO. The study of the formation of compound I under single turnover conditions and its regeneration by the addition of various salts allowed the determination of the variety of anions capable of regenerating RbMPO in the native state, i.e., I^−^, SCN^−^, Br^−^ and Cl^−^. Determination of the steady-state catalytic constants allows us to confirm that RbMPO has the same (pseudo)-halogenated substrate specificity as hMPO, i.e., SCN^−^ (1.12 × 10^5^ ± 0.31 × 10^5^ M^−1^·s^−1^) > Br^−^ (576 ± 172 M^−1^·s^−1^) > Cl^−^ (5.46 ± 0.67 M^−1^·s^−1^) [13]. The values of *k*_cat_ with SCN^−^ and Br^−^ were around 57 and 76 s^−1^, respectively. No saturation was observed towards chloride as substrate but this is the first time that a bacterial peroxidase catalyzes the formation of hypochlorous acid, the active component of bleach. Like hMPO, RbMPO has a catalase activity with *k*_cat_ catalase around 57 s^−1^. At pH 7.5 the *k*_cat_/*K*_M_ ratio was 3.33 × 10^5^ ± 1.21 × 10^5^ M^−1^·s^−1^. RbMPO has a high peroxidase activity at pH 5.5 with a *k*_2_ of 1.06 × 10^4^ ± 0.15 × 10^4^ M^−1^·s^−1^. The use of other substrates can also be considered. Iodide was effective in this study in regenerating the enzyme (Figure 5). This indicates that RbMPO has a large substrate specificity. Indeed, peroxidases are known to have an affinity for many other organic substrates such as guaiacol, ferulic acid, L-ascorbic acid, acetylsalicylic acid, etc. Bactericidal experiments on the non-pathogenic *E. coli* strain ATCC 25922 were performed in the presence of SCN^−^ and glucose oxidase as providers of H_2_O_2_. The count of surviving colonies after 1h of incubation in an enzymatic solution in the presence of 300 nM RbMPO, 25 nM GOx and 25 mM SCN^−^, confirms the results observed in kinetics at 620 nm. At these concentrations, SCN^−^ and GOx promote a halogenating activity of RbMPO, resulting in bacterial death. Biotechnological studies and biomedical applications may be considered using this enzyme as a supplier of antimicrobial compounds. The effects of temperature on the activity of the enzyme were studied for standard stopped-flow conditions. They demonstrate that RbMPO remains effective for its three (pseudo)-halogenated substrates at elevated temperatures. In the context of using the enzyme in a biomedical context and in the occurrence of bacterial infections, it is interesting to note that the enzyme of interest is active at 37 °C, which is the physiological temperature of the human body. One can also imagine the use of this enzyme on people suffering from high fever, since the enzyme is always active at 40 °C. In the same context, the study of different pH on the enzymatic activity indicates that the optimal condition is found at pH 6 in the presence of Br^−^ and at pH 7 in the presence of Cl^−^ and SCN^−^. These important results support the use of RbMPO as a microbicidal enzyme under physiological conditions in blood, urine, tears to clean contact lenses, etc. At 4 °C and 37 °C, the conservation of the enzyme in concentrated solution allows the preservation of it for at least 40 days (at 4 °C) and 20 days (at 37 °C) with activities close to 100%. The enzyme was therefore active over a wide range of temperatures and pH and was also very stable at 4 °C and 37 °C making it suitable for long-term storage and application in the human body, respectively. Finally, the bacterial enzyme permits the use of *E. coli* as a host to produce it. This is a simple and low-cost process compared to, for example, the extraction of native mammalian peroxidase from human blood.

## 5. Conclusions

**Scheme 1:**
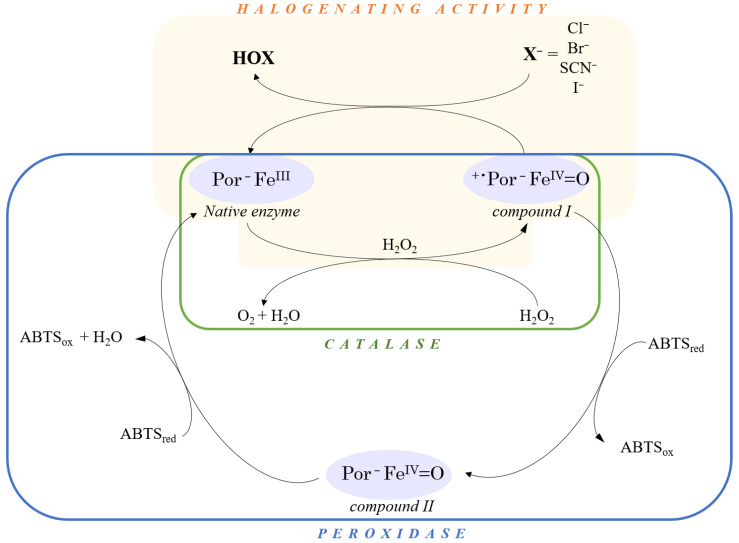
Schematic summary of the identified RbMPO activities.

In this study, a novel peroxidase was experimentally characterized using biochemical and enzymatic approaches. RbMPO was purified from inclusion bodies and reconstituted with heme, resulting in a slow conformational rearrangement around tryptophan. RbMPO catalyzes several activities as shown in Scheme 1. It catalyzes the formation of (pseudo-) hypo halogenates from I^−^, SCN^−^, Br^−^, and Cl^−^ as well as the dismutation of H_2_O_2_ and the oxidation of peroxide. RbMPO is a mammalian peroxidase homolog with high substrate specificity. A bactericidal effect with SCN^−^ as substrate and a source of H_2_O_2_ has been demonstrated. Antimicrobial development using RbMPO could be engaged.

## 6. Patent

European Patent EP22305670 “Bacterial myeloperoxidase-catalase and applications thereof” 5 May 2022 Inventors: Stines-Chaumeil Claire, Céré Claire, and Delord Brigitte. Deposited by the University of Bordeaux and CNRS. PCT/EP2023/059317.

## Figures and Tables

**Table 1:** Summarization of activity tests performed with RbMPO towards various substrates.

	Substrate 1	Substrate 2	Reagent	Detection	Wavelength nm	ε M^−1^·cm^−1^
Transition RbMPO-Fe^3+^ to Compound I or Compound I to RbMPO-Fe^3+^	H_2_O_2_	X	X	RbMPO-Fe^3+^	412	55,390
Catalase activity	H_2_O_2_	X	X	H_2_O_2_	240	43.6
Peroxidase activity	H_2_O_2_	ABTS	X	ABTS^.+^	320	36,000
Halogenase activity	H_2_O_2_	NaCl	Taurine	Taurinechlorinated	253	429
	H_2_O_2_	NaBr	Taurine	TaurineBrominated	289	415
	H_2_O_2_	NaSCN	X	SCNO^−^ formation	240	951

**Table 2:** Catalytic parameters of RbMPO at steady state determined at 37 °C in sodium phosphate buffer 50 mM pH 7.5 for catalase activity and halogenase activity at 79 µM H_2_O_2_ and pH 5.5 and 79 µM H_2_O_2_ for peroxidase activity measurement. * no saturation was observed, so the value corresponds to the ratio k_ss_/[Cl^−^] or k_ss_/[ABTS].

	Catalase Activity	Halogenase Activity			Peroxidase Activity
Substrate	H_2_O_2_	SCN^−^	Br^−^	Cl^−^	ABTS
*K*_M_ (mM)	0.17 ± 0.11	0.51 ± 0.08	133 ± 32		/
*k*_cat_ (s^−1^)	56.6 ± 20.6	56.9 ± 6.9	76.3 ± 5.12		/
*k*_cat_/*K*_M_ (M^−1^·s^−1^)	3.33 × 10^5^ ±0.21 × 10^5^	1.12 × 10^5^ ±0.31 × 10^5^	576 ± 172	5.46 ± 0.67 *	1.06 × 10^4^ ±0.15 × 10^4^ *
*K*_i_ (mM)	1.07 ± 0.7	-	-		/

## Data Availability

The data presented in this study are available on request from the corresponding author. The data are not publicly available due to the unavailability of a server dedicated to this task.

## Abbreviations

ABAH	4-aminobenzoic acid hydrazide
ABTS	2,2’-azino-bis(3-ethylbenzothiazoline-6-sulphonic acid
APF	Aminophenylfluorescein
EPO	Eosinophil peroxidase
hMPO	human myeloperoxidase
IPTG	Isopropyl β-D-1-thiogalactopyranoside
LPO	Lactoperoxidase
MMPs	Mammalian peroxidases
PDB	Protein Data Bank
RbMPO	Heme peroxidase from *Rhodopirellula baltica*
TPO	Thyroid peroxidase

## References

[1] Savelli B., Li Q., Webber M., Jemmat A., Robitaille A., Zamocky M., Mathé C., Dunand C. (2019). RedoxiBase: A database for ROS homeostasis regulated proteins. Redox Biol..

[2] Auer M., Nicolussi A., Schütz G., Furtmüller P.G., Obinger C. (2014). How Covalent Heme to Protein Bonds Influence the Formation and Reactivity of Redox Intermediates of a Bacterial Peroxidase. J. Biol. Chem..

[3] Oxvig C., Thomsen A.R., Overgaard M.T., Sørensen E.S., Højrup P., Bjerrum M.J., Gleich G.J., Sottrup-Jensen L. (1999). Biochemical Evidence for Heme Linkage through Esters with Asp-93 and Glu-241 in Human Eosinophil Peroxidase: The ester with Asp-93 is only partially formed in vivo. J. Biol. Chem..

[4] Taurog A. (1999). Molecular Evolution of Thyroid Peroxidase. Biochimie.

[5] Aratani Y. (2018). Myeloperoxidase: Its role for host defense, inflammation, and neutrophil function. Arch. Biochem. Biophys..

[6] Gan Q., Chi H., Dalmo R.A., Meng X., Tang X., Xing J., Sheng X., Zhan W. (2023). Characterization of myeloperoxidase and its contribution to antimicrobial effect on extracellular traps in flounder (*Paralichthys olivaceus*). Front. Immunol..

[7] Weiss G., Schaible U.E. (2015). Macrophage Defense Mechanisms against Intracellular Bacteria. Immunol. Rev..

[8] Schürmann N., Forrer P., Casse O., Li J., Felmy B., Burgener A.-V., Ehrenfeuchter N., Hardt W.-D., Recher M., Hess C. (2017). Myeloperoxidase Targets Oxidative Host Attacks to Salmonella and Prevents Collateral Tissue Damage. Nat. Microbiol..

[9] Krivošíková K., Šupčíková N., Gaál Kovalčíková A., Janko J., Pastorek M., Celec P., Podracká Ľ., Tóthová Ľ. (2023). Neutrophil extracellular traps in urinary tract infection. Front. Pediatr..

[10] Davies M.J., Hawkins C.L., Pattison D.I., Rees M.D. (2008). Mammalian Heme Peroxidases: From Molecular Mechanisms to Health Implications. Antioxid. Redox Signal..

[11] Sharma S., Singh A.K., Kaushik S., Sinha M., Singh R.P., Sharma P., Sirohi H., Kaur P., Singh T.P. (2013). Lactoperoxidase: Structural Insights into the Function, Ligand Binding and Inhibition. Int. J. Biochem. Mol. Biol..

[12] Ruf J., Carayon P. (2006). Structural and Functional Aspects of Thyroid Peroxidase. Arch. Biochem. Biophys..

[13] Kettle A.J., Winterbourn C.C. (2015). Chapter 12: Myeloperoxidase: Structure and Function of the Green Heme Peroxidase of Neutrophils; RCS Metallobiology. Heme Peroxidases.

[14] Fiedler T.J., Davey C.A., Fenna R.E. (2000). X-ray Crystal Structure and Characterization of Halide-Binding Sites of Human Myeloperoxidase at 1.8 Å Resolution. J. Biol. Chem..

[15] Nauseef W.M. (2018). Biosynthesis of Human Myeloperoxidase. Arch. Biochem. Biophys..

[16] Singh P.K., Iqbal N., Sirohi H.V., Bairagya H.R., Kaur P., Sharma S., Singh T.P. (2018). Structural basis of activation of mammalian heme peroxidases. Prog. Biophys. Mol. Biol..

[17] Furtmüller P.G., Burner U., Regelsberger G., Obinger C. (2000). Spectral and Kinetic Studies on the Formation of Eosinophil Peroxidase Compound I and Its Reaction with Halides and Thiocyanate. Biochemistry.

[18] Furtmüller P.G., Jantschko W., Regelsberger G., Jakopitsch C., Arnhold J., Obinger C. (2002). Reaction of Lactoperoxidase Compound I with Halides and Thiocyanate. Biochemistry.

[19] Arnhold J., Malle E. (2022). Halogenation Activity of Mammalian Heme Peroxidases. Antioxidants.

[20] Singh P.K., Ahmad N., Yamini S., Singh R.P., Singh A.K., Sharma P., Smith M.L., Sharma S., Singh T.P. (2022). Structural evidence of the oxidation of iodide ion into hyper-reactive hypoiodite ion by mammalian heme lactoperoxidase. Protein Sci..

[21] Iwamoto H., Kobayashi T., Hasegawa E., Morita Y. (1987). Reaction of Human Myeloperoxidase with Hydrogen Peroxide and Its True Catalase Activity. J. Biochem..

[22] Auer M., Gruber C., Bellei M., Pirker K.F., Zamocky M., Kroiss D., Teufer S.A., Hofbauer S., Soudi M., Battistuzzi G. (2013). A Stable Bacterial Peroxidase with Novel Halogenating Activity and an Autocatalytically Linked Heme Prosthetic Group. J. Biol. Chem..

[23] Ahariz M., Courtois P. (2010). Candida Albicans Susceptibility to Lactoperoxidase-Generated Hypoiodite. Clin. Cosmet. Investig. Dent..

[24] Cegolon L. (2020). Investigating hypothiocyanite against SARS-CoV-2. Int. J. Hyg. Environ. Health.

[25] Cegolon L., Salata C., Piccoli E., Juarez V., Palu’ G., Mastrangelo G., Calistri A. (2014). In Vitro Antiviral Activity of Hypothiocyanite against A/H1N1/2009 Pandemic Influenza Virus. Int. J. Hyg. Environ. Health.

[26] Patel U., Gingerich A., Widman L., Sarr D., Tripp R.A., Rada B. (2018). Susceptibility of Influenza Viruses to Hypothiocyanite and Hypoiodite Produced by Lactoperoxidase in a Cell-Free System. PLoS ONE.

[27] Chochola J., Yamaguchi Y., Moguilevsky N., Bollen A., Strosberg A.D., Stanislawski M. (1994). Virucidal Effect of Myeloperoxidase on Human Immunodeficiency Virus Type 1-Infected T Cells. Antimicrob. Agents Chemother..

[28] El Messaoudi K., Verheyden A.-M., Thiry L., Fourez S., Tasiaux N., Bollen A., Moguilevsky N. (2002). Human Recombinant Myeloperoxidase Antiviral Activity on Cytomegalovirus. J. Med. Virol..

[29] Denys G.A., Devoe N.C., Gudis P., May M., Allen R.C., Stephens J.T. (2019). Mechanism of Microbicidal Action of E-101 Solution, a Myeloperoxidase-Mediated Antimicrobial, and Its Oxidative Products. Infect. Immun..

[30] Musser R.C. (2011). Enhanced Lactoperoxidase System for Treatment of Milk Products. WO2011116052A3. https://patents.google.com/patent/US20110229598A1/en.

[31] Shin K., Horigome A., Yamauchi K. (2007). Oral Disinfectant, Food Additive Comprising the Disinfectant. WO2008105113A1. https://patents.google.com/patent/EP2127667A1/en.

[32] Tenovuo J., Pruitt K.M., Mansson-Rahemtulla B., Harrington P., Baldone D.C. (1986). Products of Thiocyanate Peroxidation: Properties and Reaction Mechanisms. Biochim. Et Biophys. Acta (BBA)—Protein Struct. Mol. Enzymol..

[33] Su Q., Klinman J.P. (1999). Nature of Oxygen Activation in Glucose Oxidase from Aspergillus Niger:  The Importance of Electrostatic Stabilization in Superoxide Formation. Biochemistry.

[34] Kettle A.J., Gedye C.A., Winterbourn C.C. (1997). Mechanism of Inactivation of Myeloperoxidase by 4-Aminobenzoic Acid Hydrazide. Biochem. J..

[35] Furtmüller P.G., Obinger C., Hsuanyu Y., Dunford H.B. (2000). Mechanism of reaction of myeloperoxidase with hydrogen peroxide and chloride ion. Eur. J. Biochem..

